# Retraction: 17β-estradiol upregulates striatin protein levels via Akt pathway in human umbilical vein endothelial cells

**DOI:** 10.1371/journal.pone.0318041

**Published:** 2025-01-17

**Authors:** The PLOS One Editors

Following the publication of this article [1], concerns were raised regarding results presented in Figs 3, 4, 5, and 6. Specifically,

- The following panels in Figs 3C, 4C and 6 appear to partially overlap, despite being used to present different experimental conditions:
  ○ Fig 3C Striatin siRNA1, Fig 4C PL-con, Fig 6 Scrambled CON, and Fig 6 siRNA1 CON.
  ○ Fig 3C Striatin siRNA3 and Fig 4C PL-con
  ○ Fig 4C PL-striatin and Fig 6 Scrambled E2
  ○ Fig 6 siRNA1 E2 and Fig 6 siRNA2 E2
  ○ Fig 6 siRNA1 CON, Fig 6 siRNA2 CON, and Fig 6 siRNA3 CON.
- The following panels in Figs 3B, 4B and 5 appear to partially overlap, despite being used to present different experimental conditions:
  ○ Fig 3B Scrambled siRNA 0h and Fig 3B Striatin siRNA1 0h
  ○ Fig 3B Striatin siRNA2 0h and Fig 5 siRNA1 CON 0h
  ○ Fig 3B Striatin siRNA3 0h and Fig 4B PL-striatin 0h
  ○ Fig 3B Striatin siRNA3 0h and Fig 4B PL-con 0h
  ○ Fig 4B PL-con 0h and Fig 5 siRNA3 E2 0h
  ○ Fig 3B Striatin siRNA3 24h and Fig 5 Scrambled CON 24h
  ○ Fig 5 siRNA1 CON 24h and Fig 5 siRNA2 E2 24h
  ○ Fig 5 siRNA CON 24h and Fig 5 siRNA3 CON 24h

In light of the above concerns that question the integrity and reliability of the published results, the *PLOS One* Editors retract this article.

SZ, PS, and JX did not agree with the retraction. HL, RL, LL, YX, and SC either did not respond directly or could not be reached.
